# Cardiometabolic Phenotypic Differences in Male Offspring Born to Obese Preeclamptic-Like BPH/5 Mice

**DOI:** 10.3389/fped.2021.636143

**Published:** 2021-09-22

**Authors:** Kalie F. Beckers, Viviane C. L. Gomes, Kassandra J. Raven Crissman, Daniella M. Adams, Chin-Chi Liu, Fabio Del Piero, Scott D. Butler, Jenny L. Sones

**Affiliations:** ^1^Veterinary Clinical Sciences, School of Veterinary Medicine, Louisiana State University, Baton Rouge, LA, United States; ^2^Pathobiological Sciences, School of Veterinary Medicine, Louisiana State University, Baton Rouge, LA, United States; ^3^Biomedical Sciences, College of Veterinary Medicine, Cornell University, Ithaca, NY, United States

**Keywords:** preeclampsia, fetal programming, obesity, sex differences, adiposity

## Abstract

Preeclampsia (PE) is a hypertensive disorder of pregnancy occurring in approximately 10% of women worldwide. While it is life threatening to both the mother and baby, the only effective treatment is delivery of the placenta and fetus, which is often preterm. Maternal obesity is a risk factor for PE, and the effects of both on offspring are long standing with increased incidence of cardiometabolic disease in adulthood. Obese BPH/5 mice spontaneously exhibit excessive gestational weight gain and late-gestational hypertension, similar to women with PE, along with fetal growth restriction and accelerated compensatory growth in female offspring. We hypothesized that BPH/5 male offspring will demonstrate cardiovascular and metabolic phenotypes similar to BPH/5 females. As previously described, BPH/5 females born to *ad libitum*-fed dams are overweight with hyperphagia and increased subcutaneous, peri-renal, and peri-gonadal white adipose tissue (WAT) and cardiomegaly compared to age-matched adult female controls. In this study, BPH/5 adult male mice have similar body weights and food intake compared to age-matched control mice but have increased inflammatory subcutaneous and peri-renal WAT and signs of cardiovascular disease: left ventricular hypertrophy and hypertension. Therefore, adult male BPH/5 do not completely phenocopy the cardiometabolic profile of female BPH/5 mice. Future investigations are necessary to understand the differences observed in BPH/5 male and female mice as they age. In conclusion, the impact of fetal programming due to PE has a transgenerational effect on both male and female offspring in the BPH/5 mouse model. The maternal obesogenic environment may play a role in PE pregnancy outcomes, including offspring health as they age.

## Introduction

Preeclampsia (PE) is characterized by maternal hypertension occurring after 20 weeks of gestation (systolic ≥140 mmHg or diastolic ≥90 mmHg) along with another accompanying sign/symptom, including proteinuria, renal insufficiency, thrombocytopenia, hepatic dysfunction, and/or pulmonary edema (1). PE affects up to 300,000 women worldwide, making it a leading cause of maternal and fetal morbidity and mortality (2). The treatment is often delivery of the fetus and the placenta, which can have deleterious consequences on both the mother and baby (3). Offspring are often premature or stillborn, exhibit fetal growth restriction (FGR), and are small for gestational age (2). Beyond the perinatal effects of being born to PE mothers, the lifelong consequences can be devastating for the offspring. Fetal programming, in-*utero* alterations occurring due to the maternal environment, can affect fetal growth and development leading to lifelong effects on the offspring (4). PE-associated fetal programming can result in increased cardiovascular complications, including hypertension, ischemic heart disease, and stroke (5), and metabolic disease in the offspring as they age (1, 6, 7). PE outcomes are in line with the Developmental Origins of Health and Disease theory, by demonstrating that an unfavorable uterine environment will lead to pathogenic conditions in the offspring, which will increase the risk of chronic disease later in life (8). Furthermore, poor prenatal nutrition is associated with low birth weights, which are linked to changes in adult body composition, including altered fat distribution, reduced muscle mass, and low bone mineral content (4). One theory is that in response to an adverse intrauterine environment, the fetus adapts for survival. Fetal responses include altered metabolic homeostasis, downregulation of growth, and endocrine changes (9, 10). These adaptive changes may be beneficial to the fetus short term, while long term they are detrimental to the offspring health. Changes include compensatory growth, diet-induced obesity, hyperphagia, and other factors (10, 11).

Obesity has reached epidemic proportions in majority of the developed world, with over 50% of women of childbearing age being overweight or obese (12). In 2018, over 40% of the men in the United States were considered obese (13). Previous research links maternal and early life nutrients to the development of long-term metabolic disorders including alteration to organogenesis, tissue development, and metabolism. These changes predispose offspring to obesity and metabolic and cardiovascular diseases later in life (14–16). There are both human and animal models that have evidence linking maternal obesity to “programming” the offspring to develop cardiometabolic disease in adulthood (17–21). It has also been determined that the adverse fetal programming is more pronounced with obesity vs. malnutrition (22). The BPH/5 mouse model, originally described by (23), spontaneously develops PE. Therefore, it is possible to study the generational effects of PE using BPH/5 mice. Specifically, BPH5 females have mildly elevated blood pressure prior to pregnancy, which is a known risk factor for the development of PE in women (23). In late gestation, the dams display elevated mean arterial pressure (MAP) and proteinuria, endothelial dysfunction, and renal glomerulosclerosis (23). Similar to PE in women, BPH/5 offspring have lower birth weights compared to normotensive C57 control mice, indicative of FGR, and litter size is severely compromised due to fetal demise. BPH/5 show placental abnormalities, such as upregulation of pro-inflammatory mediators (24, 25).

Most of the cardiovascular outcome studies focus on women/female offspring born to PE mothers because of the transgenerational effect they play in the life cycle of PE. This study aims to examine the importance of PE fetal programming on male offspring in the BPH/5 model. A previous research by Sutton et al. studied the effects of in-*utero* PE on the female offspring of obese BPH/5 (26). Female BPH/5 offspring are born smaller than C57 controls and exhibit excessive catch up growth and hyperphagia. Sutton et al. also demonstrated that BPH/5 female mice have a predisposition for obesity with increased white adipose tissue (WAT) accumulation, pro-inflammatory reproductive WAT, and leptin dysfunction (26). We hypothesized that male BPH/5 offspring born to PE-like dams will demonstrate cardiovascular and metabolic phenotypes similar to BPH/5 females.

## Materials and Methods

### Animal Experiments

The Louisiana State University Institutional Animal Care and Use Committee approved all animal experiments. Body weights were assessed in prepubertal (2–3 weeks) BPH/5 (*n* = 10) and C57 (*n* = 15) and adult (8 weeks−6 months) BPH/5 (*n* = 32) and C57 (*n* = 17). All males within a given litter from at least three litters were used for animal experiments. Normal chow [Purina (Neenah, WI) rodent chow: 23% crude protein, 4.5% crude fat, 6% crude fiber, and 8% ash] food intake was measured for 12 consecutive days from adult C57 and BPH5 male mice after 2 days of accumulation. Using a gram scale, body weights, visceral WAT (peri-gonadal and peri-renal depots), inguinal subcutaneous WAT, subscapular brown adipose tissue (BAT), hearts with left ventricle and LV dissected, kidneys, and livers were weighed from BPH/5 and C57 age-matched adult males. All WAT and BAT depots were dissected free from surrounding tissues according to published methods in mice (27). Tissues were flash frozen in liquid nitrogen for downstream analyses.

### Radiotelemetric Measurement of Blood Pressure and Heart Rate

Adult male BPH/5 (*n* = 4) and C57BL/6 (*n* = 16) mice underwent carotid implantation of telemetry (Data Sciences International) according to published methods (23). Briefly, male mice were anesthetized for placement of a telemeter in the left carotid artery and transmitter body in the subcutaneous space. Mice were allowed to recover for 10 days, followed by 4 days of heart rate (beats per min) and MAP recording.

### Histology

The heart, kidney, liver, and peri-renal and subcutaneous WAT were fixed in 10% formalin, paraffin-embedded sectioned, and stained using hematoxylin and eosin (H&E) by the Louisiana Animal Disease Diagnostic Laboratory standards and analyzed by a board-certified veterinary pathologist. A blinded single operator measured adipocyte area in six randomly selected frames per mouse (*n* = 3/strain) using ImageJ (NIH).

### Quantitative PCR

Total RNA was extracted from peri-renal and inguinal subcutaneous WAT using TRIzol according to manufacturer's instructions (Qiagen, Hilden, Germany). RNA quality and quantity were assessed by spectrophotometry (NanoDrop). Using the qScript cDNA kit (Quanta BioSciences, Beverly, MA), 1,000 ng cDNA was reverse transcribed. Each quantitative PCR (qPCR) was performed in triplicate with an ABI 7,500 Fast Thermocycler (Applied Bioscience) using SYBR Green (Quanta BioSciences) using 25 ng cDNA. The following forward and reverse primers were used, respectively: *TNFa* [GAACTGGCAGAAGAGGCACT and AGGGTCTGGGCCATAGAACT (26)], *IL-6* [TGGCTAAGGACCAAGACCATCCAA and AACGCACTAGGTTTGCCGAGTAGA (26)], and *Ptgs-2* [ACTGGGCCATGGAGTGGACTTAAA and AACTGCAGGTTCTCAGGGATGTGA (25)] expression level. Data was analyzed using the ΔΔ Ct method, and results were normalized to *18s* [CCGGGCTTCTATTTTGTTGGT and TAGCGGCGCAATACGAATG (28)] (29).

### Statistical Analysis

Statistical analysis was performed using GraphPad Prism. Shapiro–Wilk test was used to check for normality. Two-way ANOVA with Tukey's *post hoc* test and/or a Student's *t*-test were used. The *p* values < 0.05 are considered significant. Error bars within figures were recorded in standard error mean (SEM).

## Results

### Adult BPH/5 Male Body Weight and Food Intake Are Similar to Control Male Mice, While Visceral and Subcutaneous Adipose Tissue Depots Are Increased

**Figure 1:**
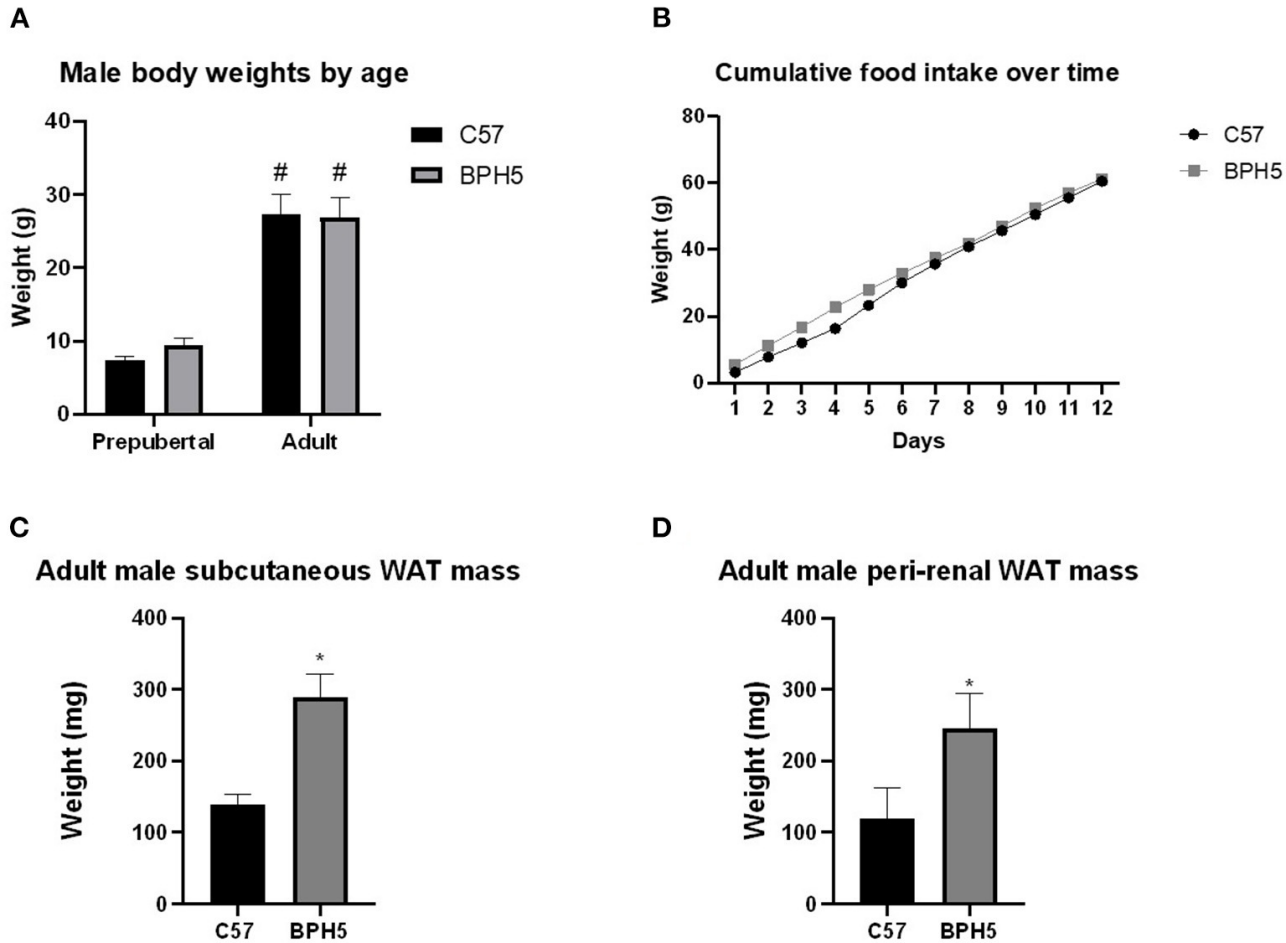
Adult BPH/5male phenotypic differences in body weight, food intake, and white adipose tissue (WAT). **(A)** Body weights were measured in prepubertal and adult BPH/5 males, and a similar weight was demonstrated at both prepubertal and adulthood when compared to C57 males (*n* = 10–32/group). **(B)** Daily and cumulative food intake was measured for 12 days in both BPH5 and C57 male and was found to be not significantly different (*n* = 5/group). **(C)** BPH5 males have significantly increased subcutaneous WAT (*n* = 6–18/group). **(D)** BPH/5 males have more peri-renal WAT vs. C57 (*n* = 8/group) (^*^p < 0.05 vs. C57, ^#^*p* < 0.05 vs. prepubertal weights of their respective strain).

In humans, infants with low birth weights have been shown to exhibit accelerated catch up growth and central pattern of fat distribution, reduced lean mass, and increased adiposity (30, 31). Offspring of preeclamptic-like BPH/5 dams have previously been described to have intrauterine FGR and have smaller birthweights when compared to C57 aged-matched counterparts (23, 25, 32). It was previously described that female BPH/5 offspring exhibit accelerated catch up growth beginning with small for gestational age birth weights, then overweight by adulthood when compared to C57 female controls (12). In this study, male BPH5 offspring have similar prepubertal and adult body weights when compared to C57 age-matched controls (Figure 1A; *p* > 0.05), while male mice in both strains have significantly increased body weight from the prepubertal stage into adulthood (Figure 1A; *p* < 0.05). BPH/5 adult female mice are hyperphagic when compared to adult C57 female mice (26). However, BPH/5 adult male mice show daily food intake and cumulative food intake over 12 days (Figure 1B) that are comparable to adult C57 male mice (*p* > 0.05). Similar to findings in Sutton et al., BPH/5 males exhibited increased WAT mass in the subcutaneous (Figure 1C) and peri-renal depots (Figure 1D). This was not associated with an increase in BPH/5 adipocyte area when measured histologically after H&E staining compared to age-matched C57 adult males (data not shown). Peri-gonadal WAT was not significantly different in BPH/5 males compared to C57 aged-matched controls (*p* > 0.05; Supplementary Table 1) as was also found in BPH/5 adult females. Furthermore, intrascapular BAT weights were not different when compared to controls (Supplementary Table 1).

### Adult BPH5 Male Mice Show Evidence of Cardiovascular Disease

**Figure 2:**
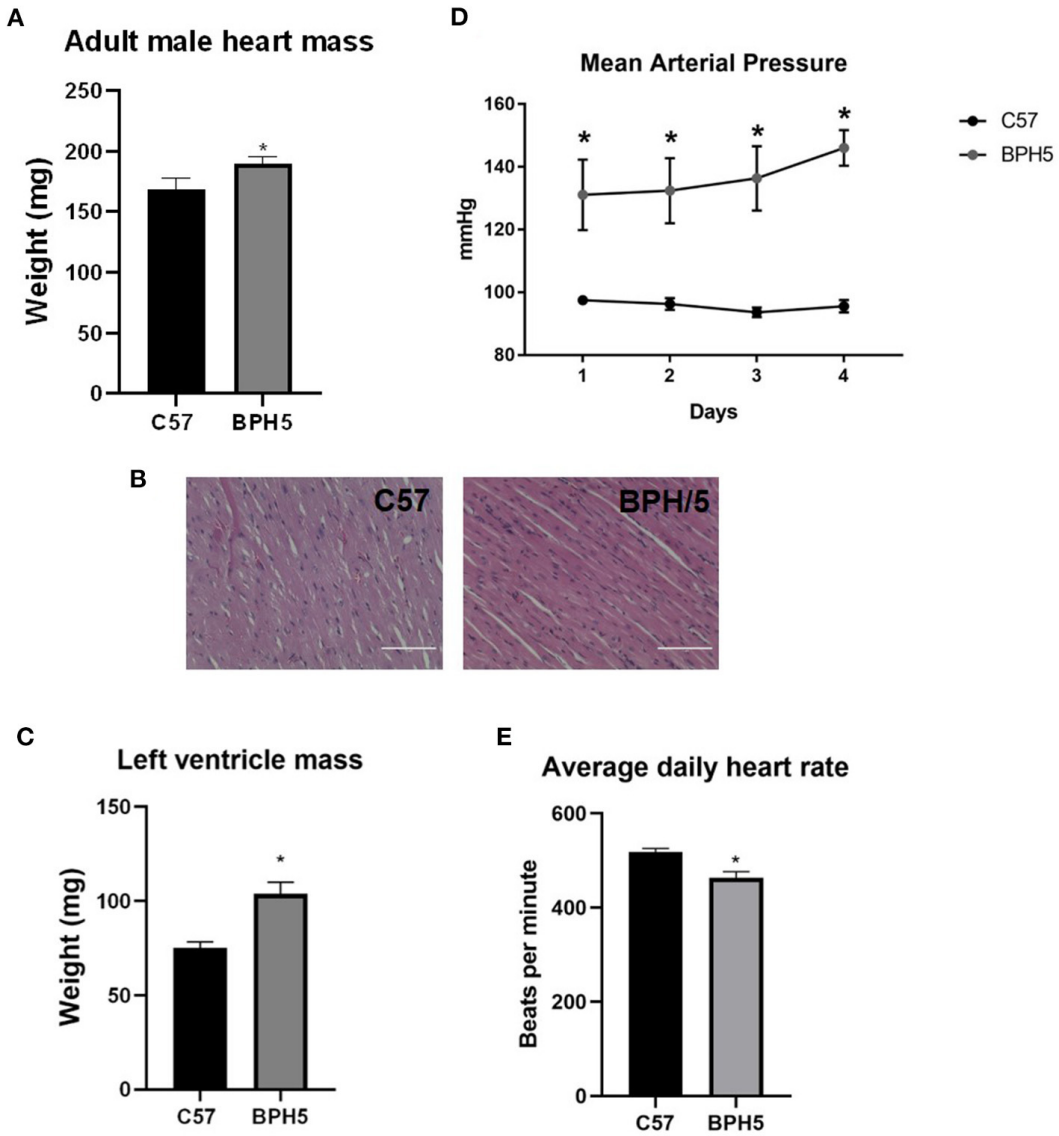
Adult BPH/5 male mice exhibit signs of cardiovascular disease. **(A)** BPH5 male mice demonstrate increased heart mass compared to C57 controls. **(B)** Hematoxylin and eosin (H&E) stain of C57 (left) and BPH/5 (right) cardiac tissue. Scale bar = 100 μm. **(C)** BPH5 male mice demonstrate increased mass of the left ventricle compared to C57 controls. **(D)** BPH5 male mice demonstrate increased mean arterial pressure compared to C57 controls as measured over 4 consecutive days. **(E)** Daily average heart rates measured as beats per min were increased in BPH/5 adult males compared to C57 (*n* = 4–18/group; *p < 0.05 vs. C57).

Low birth weights in humans have been associated with development of cardiovascular disease (4–6, 31). The adult male BPH5 offspring have increased heart weights compared to age-matched C57 mice, indicative of cardiomegaly (Figure 2A; *p* < 0.05). On histological examination, BPH/5 exhibit mild abnormal fiber size variation with variously sized occasional larger nuclei (karyomegaly). There were also rare areas of cardiomyocyte necrosis with hypereosinophilic cytoplasm and pyknotic nuclei (Figure 2B). This evidence for cardiomyopathy with mild acute ischemic necrosis in BPH/5 adult males was not identified in age-matched C57 adult males (Figure 2B). Cardiomegaly in adult BPH/5 males is accompanied by increased left ventricle mass as compared to age-matched control C57 mice (Figure 2C; *p* < 0.05). Mean arterial pressure in adult BPH/5 males is increased compared to age-matched control C57 mice (Figure 2D; *p* < 0.05), while average daily heart rates are decreased (Figure 2E; *p* < 0.05). The BPH5 male offspring also displayed increased liver weight compared to controls (17.8%), suggestive of hepatomegaly (Supplementary Table 1; *p* < 0.05). Finally, adult male BPH5 offspring have increased kidney weight compared to adult male C57 controls (33%), suggestive of nephromegaly (Supplementary Table 1; *p* < 0.05). However, on histological examination, no significant lesions were identified in the liver nor the kidney.

### Inflammatory Mediators Are Increased in BPH5 Male White Adipose Tissue Depots

**Figure 3:**
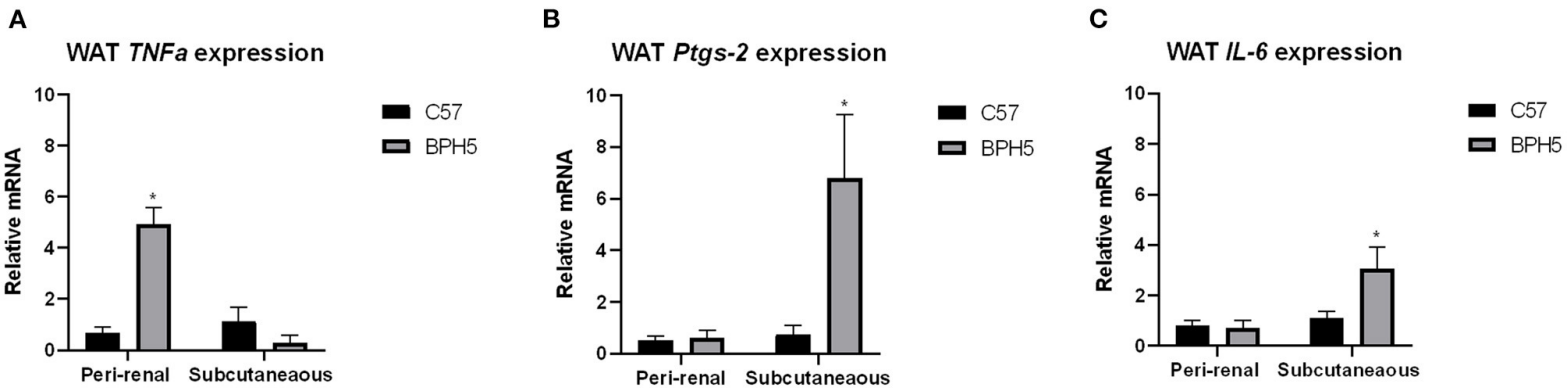
Adult BPH/5 male mice exhibit increased inflammatory mediators within peri-renal and subcutaneous white adipose tissue (WAT). **(A)** Using real-time PCR, adult BPH/5 males have significantly increased tumor necrosis factor alpha (*TNFa*) relative mRNA expression in the peri-renal WAT; **(B)** prostaglandin synthase 2 (*Ptgs-2*) relative mRNA expression was significantly increased in subcutaneous WAT; and **(C)** interleukin-6 (*IL-6*) mRNA expression was significantly increased in the subcutaneous WAT (*n* = 3–8/group; *p < 0.05 vs. C57).

Increased adiposity is linked to increased adipose tissue inflammation with upregulation of inflammatory cytokines (33). Reproductive WAT of adult BPH/5 female offspring displayed a seven- and four-fold increase in tumor necrosis factor alpha (*TNFa*) and interleukin-6 (*IL-6*) mRNA, respectively (26). Adult BPH5 males showed an increase in *TNFa* relative mRNA in the peri-renal but not the subcutaneous WAT compared to age-matched C57 adult males (Figure 3A; *p* < 0.05). In subcutaneous WAT, there was a significant difference in prostaglandin synthase 2 (*Ptgs-2*) (Figure 3B) and *IL-6* mRNA expression (Figure 3C) compared to adult male C57 controls (*p* < 0.05).

## Discussion

PE during pregnancy can be life threatening for both the mother and fetus, and cardiometabolic comorbidities may persist long term. The BPH5 mouse model, which spontaneously develops PE, has been used to gain insight into the pathophysiology and outcomes of PE. This study focuses on the male offspring phenotype when exposed in *utero* to a maternal obesogenic environment. Genetic influence is important to take into consideration when analyzing the differences between offspring outcomes.

Differences in the pathophysiology of PE may occur depending on the sex of the fetus. For example, maternal endothelial dysfunction, characterized by peripheral microvascular vasoconstriction, was greater in preeclamptic pregnancies carrying a male fetus (34). The normal postnatal growth of male neonates from preeclamptic pregnancies suggests that fetal–placental blood flow is maintained despite maternal hypertension and placental insufficiency (34). There is still a need for further studies examining the effects of placental insufficiency on postnatal body composition, specifically adipose tissue distribution, type, and glucose homeostasis. For male infants, those born to preeclamptic women had greater microvasculature blood flow at 6 h postnatal while male infants of normotensive women exhibited increasing blood flow with time (34). It was also found that the PE-driven maternal endothelial dysfunction was greater in the presence of a male fetus (35).

Solely studying this disease in humans presents various challenges, such as inability for *in vivo* manipulative studies. The spontaneously hypertensive rat (SHR) is a model of essential hypertension in humans and one in which males have higher blood pressure (BP) than females as young adults (36), but both sexes are hypertensive compared with normotensive Sprague–Dawley rats. A rat model of placental ischemia, which occurs in PE, is the reduced uterine perfusion pressure (RUPP) model, and offspring of RUPP females exhibit intrauterine FGR (37, 38), as is also commonly found in offspring of women who develop PE. The male offspring of a RUPP pregnancy show increased BP with adulthood, whereas the female offspring do not (39). According to Reckelhoff et al., males demonstrate higher BP than females in hypertensive rat models (40). Similar differences may occur in these BPH5 offspring, contributing to the female showing obesity while the males apparently do not. Thus, investigating PE-driven cardiometabolic outcomes in male offspring is equally important as female offspring.

An unfavorable maternal environment, including obesity as well as malnutrition, has been shown to impact offspring into adulthood. The Dutch Winter famine, which occurred during World War II when food was limited, resulted in offspring having increased abdominal fat distribution in adult male (41), demonstrating fetal programming likely occurred because of maternal undernutrition. The fetuses from the famine were programmed in *utero* to have an altered fat distribution and metabolism. The offspring from the Dutch Famine cohort had a three-fold increase of coronary heart disease (42). As previously mentioned, the effects of maternal obesity are more pronounced than malnutrition (22). This may be comparable to the BPH5 offspring where the obese mother is an unfavorable environment for offspring development. This may lead to adverse offspring outcomes, including increased adiposity and cardiovascular disease.

BPH5 preeclamptic mouse model are known to have offspring with low birth weights (23). The BPH5 female offspring showed post pubertal accelerated catch up growth with adulthood obesity (26). Interestingly, male BPH5 offspring may demonstrate an earlier accelerated catch up growth as BPH5 males catch up to C57 male body weight by 2–3 weeks of age. Accelerated catch up growth has been associated with increased adiposity in both adult male or female rats (43). Furthermore, accelerated catch up growth has also been associated with hyperphagia, leading to hyperleptinemia, hypertension, and obesity in adulthood, which is similar to BPH5 females (26, 44). Therefore, we hypothesized and found that BPH/5 males do not demonstrate hyperphagia as females do and, unlike BPH/5 females, maintain similar body weights to male C57 mice from the prepubertal stage and into adulthood. Studies investigating the lactational feeding period in BPH/5 would be of interest as postnatal overfeeding has been associated with hyperphagia and obesity later in life in both sexes (45, 46). Bol et al. demonstrated that in overfed lactating mice, the male offspring showed an increased fat mass compared to controls (47).

Another contributing factor to the increased adiposity could be associated with developmental changes in the population of cells in the adipose tissue. Obesity can result from expansion of fat mass due to adipocyte hypertrophy by lipid accumulation or an exaggerated number of adipocytes (48). Offspring from diet-induced obese mothers showed increased fat mass with larger adipocytes in adulthood (46). The BPH5 male offspring demonstrated increased peri-renal and subcutaneous WAT mass, but not in adipocyte area, and similar amounts of BAT when compared to age-matched controls. It has been shown that an early reduction in BAT may perpetuate through the life cycle and suppress energy expenditure, therefore promoting increased WAT and obesity (49). Therefore, BPH5 males that do not demonstrate a reduced BAT indicate that they may not exhibit the full obesogenic profile as their female counterparts do. In the newborn, the majority of the adipose tissue is BAT, which is used for thermoregulation in the extra-uterine environment (49). Adipose tissue first appears during mid-gestation and increases through late gestation, then becomes a mixture of both BAT and WAT. After birth, majority of the BAT depot becomes WAT. The amount, location, and type of adipose tissue is affected by multiple factors, which all play a role in the glucose homeostasis of the offspring (49). It has been shown that an early reduction in BAT can suppress energy expenditure and promote obesity (49). Further investigations into BAT function in BPH/5 males are ongoing.

**Figure 4:**
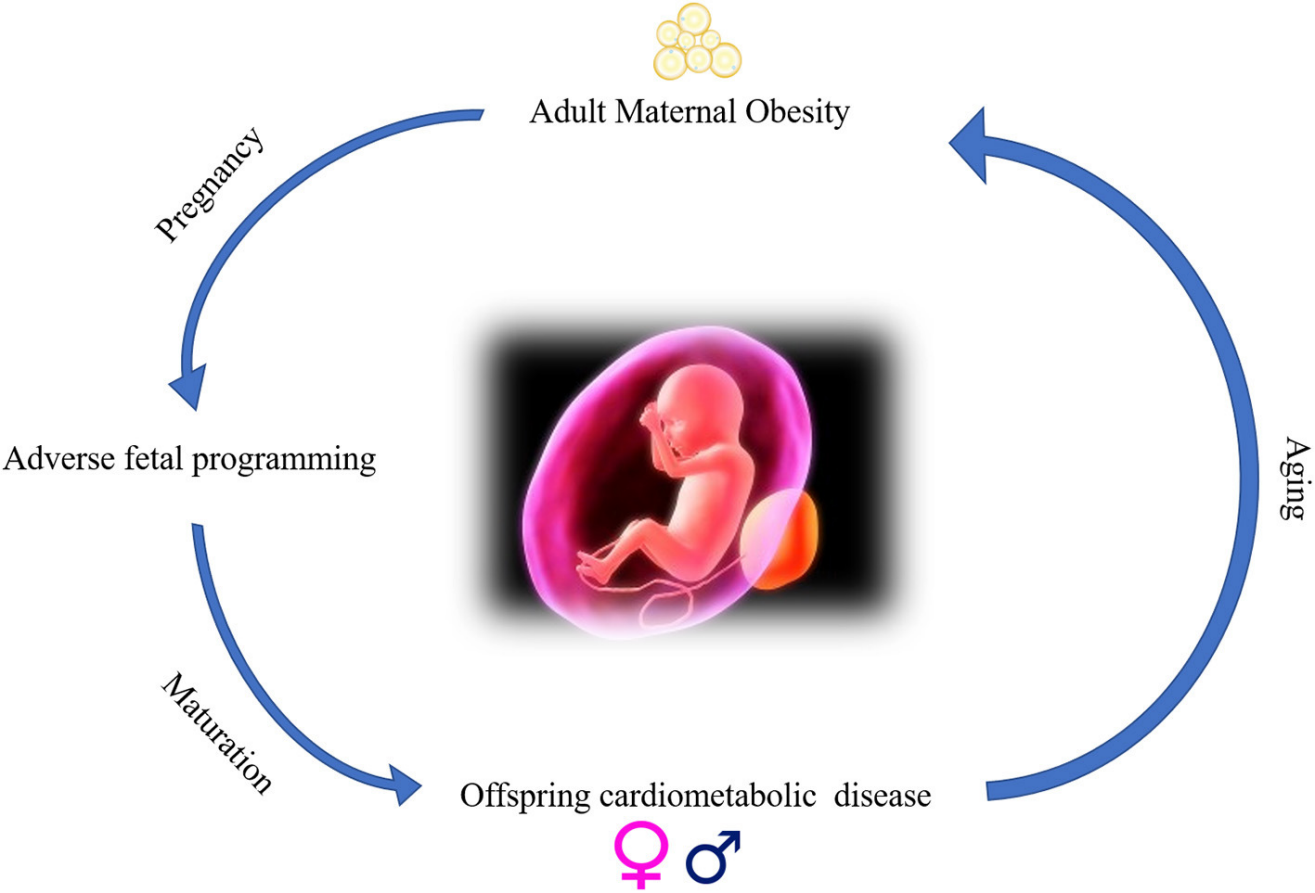
Working hypothesis for effects of maternal obesity on fetal programming. A depiction of the in-*utero* effects of maternal obesity on offspring demonstrated by BPH5 mouse model. Adverse fetal programming attributing to low birth weight and early accelerated catch up growth. Offspring cardiometabolic disease accrediting to increased adiposity and cardiomegaly found in this study. Image made with Creative Commons.

Similarly to the unfavorable intrauterine environment of PE, infants born to diabetic mothers have been shown to demonstrate more adipose tissue when compared to controls (50). Fetal programming from obese mothers has been shown to result in hyperphagia, changes in cellular composition, lower energy expenditure, or modification of the neurohormonal axis (50, 51). Thus, it is possible that fetal programming from an adverse maternal environment in this model could promote a pro-inflammatory milieu in the WAT, which may favor the development of the cardiovascular disease in this mouse model. Human and animal studies have shown that obesity is associated with low-grade, chronic inflammation in the adipose tissue (52). Inflammation with the adipose tissue has been found to be a key contributor to the pathogenesis of metabolic syndrome and other cardiovascular disease (53, 54). It has also been linked to insulin resistance and type 2 diabetes (55, 56). TNFa and IL-6 are produced and secreted by adipocytes and may be related to beta cell function (57). They are also produced by immune cells in the WAT, including adipose tissue macrophages (58). Ptgs-2 in WAT has also been associated with beta cell dysfunction and increased oxidative stress (57). A high fat diet fed to C57 mice to mimic obesity induced an upregulation in *IL-6, TNFa*, and *Ptgs-2* gene expression from adipose tissue (52). Increased inflammation of visceral adipose tissue might lead to an increase in the delivery of free fatty acids to the liver, contributing more to the development of obesity (57). In the BPH5 male mice, *TNFa* and *Ptgs-2* were increased similar to the studies above, suggesting that these male mice may exhibit inflamed adipose tissue with oxidative stress that may contribute to cardiometabolic disease. Although there were no histological signs of liver disease, pro-inflammatory visceral and subcutaneous WAT depots in adult male BPH/5 mice may promote progressive liver disease as they age. These long-term studies in BPH/5 male and female mice would be valuable to better understand the chronic effects of maternal obesity-associated fetal programming as offspring age (Figure 4).

BPH5 offspring demonstrate a cardiometabolic phenotype with markers such as central obesity and cardiovascular disease in the females. The males exhibit a unique phenotype showing increased visceral and subcutaneous adiposity and cardiovascular disease. In summary, BPH5 males contrast the females as the female offspring strongly demonstrate the obese metabolic phenotype. In conclusion, maternal obesity and altered fetal programming may play a role in these sex-dependent offspring outcomes into adulthood.

## Data Availability Statement

The raw data supporting the conclusions of this article will be made available by the authors, without undue reservation.

## Ethics Statement

The animal study was reviewed and approved by Louisiana State University Institutional Animal Care and Use Committee.

## Author Contributions

KB, VG, KC, DA, C-CL, SB, and JS: data collection and analysis. KB, VG, C-CL, FP, and JS: data interpretation. KB and JS: initial manuscript draft. KB, VG, KC, DA, C-CL, SB, FP, and JS: manuscript edits and final approval. All authors have read and agreed to the published version of the manuscript.

## Funding

This study received funding from the National Institutes of Health P20GM135002 (JS).

## Conflict of Interest

The authors declare that the research was conducted in the absence of any commercial or financial relationships that could be construed as a potential conflict of interest.

## Publisher's Note

All claims expressed in this article are solely those of the authors and do not necessarily represent those of their affiliated organizations, or those of the publisher, the editors and the reviewers. Any product that may be evaluated in this article, or claim that may be made by its manufacturer, is not guaranteed or endorsed by the publisher.
