# Supplementary material for: Cardiometabolic Phenotypic Differences in Male Offspring Born to Obese Preeclamptic-Like BPH/5 Mice
Source: Front Pediatr. 2021 Sep 22;9:636143. doi: 10.3389/fped.2021.636143 (PMC8493471; doi:10.3389/fped.2021.636143)
Supplement: Supplementary file 1 [file Data_Sheet_1.PDF]

Table 1. Kidney, liver, peri-gonadal white adipose tissue, and brown adipose tissue mass of BPH/5 and C57 adult male mice

**Tissue Mass (mg)**	**C57**	**BPH/5**	**% difference**	**P value**
**Kidney**	414	551	33.09 %	0.0014 *
**Liver**	1230	1450	17.8 %	0.0068 *
**Peri-gonadal white adipose tissue (WAT)**	434.4	470.1	8.2 %	0.34
**Brown adipose tissue (BAT)**	152.3	163.1	7.09 %	0.57
